# Acute disc herniation following surgical decompression of lumbar spinal stenosis: a retrospective comparison of mini-open and minimally invasive techniques

**DOI:** 10.1186/s13018-023-04457-2

**Published:** 2023-12-18

**Authors:** Ofir Uri, Liad Alfandari, Yoram Folman, Amit Keren, William Smith, Inbar Paz, Eyal Behrbalk

**Affiliations:** 1 Spine Surgery Unit, Orthopedic Department, Hillel Yaffe Medical Center, Ha-Shalom, 38100 Hadera, Israel; 2 Department of Neurosurgery, University Medical Center, Las Vegas, NV USA

**Keywords:** Lumbar spinal stenosis, Minimally invasive spine decompression, Mini-open spine decompression, Postoperative disc herniation, Lumbar instability

## Abstract

**Background:**

Disc herniation following decompression of lumbar spinal stenosis is a less familiar surgical complication. Previous studies suggested that open lumbar decompression techniques, associated with relative segmental instability especially in the presence of degenerated disc in older patients, are more likely to result in disc herniation compared to minimally invasive techniques. The current study compares the incidence of acute disc herniation following mini-open and minimally invasive decompression of lumbar spinal stenosis.

**Methods:**

This was a retrospective study reviewing 563 patients who underwent spinal decompression for symptomatic lumbar stenosis by mini-open bilateral partial laminectomy technique or minimally invasive laminotomy utilizing a tubular system. Demographic and clinical data were collected and compared between the groups.

**Results:**

Postoperative disc herniation rate was significantly lower in the minimally invasive group with 2 of 237 cases (0.8%) versus 19 of 326 cases (5.8%) in the mini-open group (*p* = 0.002). This finding was more noticeable following multi-level procedures with no case of postdecompression disc herniation in the minimally invasive group compared to 8 of 39 cases (20.5%) in the mini-open group (*p* = 0.003).

**Conclusion:**

The incidence of postoperative disc herniation following spinal decompression for symptomatic lumbar stenosis was 5.8% following mini-open bilateral partial laminectomy compared to only 0.8% after minimally invasive laminotomy (*p* = 0.002). These findings highlight the more extensive nature of mini-open surgery associated with relative segmental instability that poses a greater risk for postoperative disc herniation.

## Background

Lumbar spinal stenosis (LSS) is a common diagnosis, evident radiographically in 19–47% of patients over the age of 60 years and clinically symptomatic in about 10% [1, 2]. Decompression surgery is indicated for patients with moderate to severe symptoms, refractory to non-operative treatment [3, 4]. Many surgical techniques of LSS decompression are available, ranging from traditional open laminectomy, mini-open partial laminectomy and different minimally invasive surgical techniques involving less bone resection and soft tissue damage [3–7]. Minimally invasive spinal decompression is associated with a shorter hospital stay and less blood loss compared to open decompression techniques [5–7] and with a lower risk of developing segmental instability at the affected level due to less extensive surgical exposure and less bone resection [8–11]. Nevertheless, most studies present similar clinical outcomes and major complication rate of minimally invasive and open decompression techniques of LSS and the literature remains inconclusive regarding the superiority of one technique over the other [5–7]. Several studies described a less familiar complication of acute disc herniation following LSS decompression, but did not compare its rate between different surgical techniques [12, 13]. The higher incidence of segmental instability associated with open LSS decompression in the presence of a degenerated disc with frail annulus (common in elderly patients) was suggested as a risk factor of disc herniation following LSS decompression [12].

We hypothesized that decompression of LSS by minimally invasive technique using a tubular system with less bone and soft tissue damage will result in a lower incidence of postoperative disc herniation compared to mini-open decompression technique. To our knowledge, no previous study compared the rate of this less familiar complication between these two surgical techniques.

## Methods

### Study design

A retrospective, comparative study analyzed data of 662 patients who underwent primary lumbar decompression for symptomatic LSS in our institution between February 2015 and September 2020. The study was approved by our institutional ethical board. Clinical and demographic data (gender, age, BMI, surgical approach, affected level) were collected retrospectively from the hospital medical records.

### Patient population

All patients who underwent surgical decompression for symptomatic LSS in our spine surgery unit by the senior author (E.B) were candidates for inclusion in the study. The patients underwent full clinical evaluation and physical examination in our outpatient clinic. Preoperative CT and MRI imaging of the lumbar spine was obtained in all cases. All patients were diagnosed with intractable radicular pain and/or neurogenic claudication with disability related to LSS for at least 6 months, irresponsive to non-operative treatment. MRI imaging of all patients demonstrated severe to extreme spinal stenosis as described by Schizas et al. [14]. Patients with mild to moderate lumbar stenosis were treated non-operatively. All patients were followed up for a minimum of 12 months postoperatively. We excluded patients who had previous spinal surgery or pre-existing disc herniation at the operated level. Degenerative grade I spondylolisthesis was not a contraindication for decompression without instrumentation if no signs of spinal instability were seen on dynamic flexion–extension radiographs.

### Surgical technique and follow-up

All patients were operated on by the senior author (E.B), a specialized followship-trained spine surgeon, and underwent lumbar decompression for their symptomatic LSS through a mini-open midline approach (from February 2015 to March 2018) or a minimally invasive approach utilizing a tubular system (from April 2018 to September 2020).

#### Mini-open approach

Under general anesthesia, patients were placed prone on Jackson table. Surgical level was identified under fluoroscopy. A 4–5-cm midline longitudinal incision was performed, and subperiosteal dissection was carried out using McCulloch retractor for soft tissue retraction until reaching the interlaminar gap. Using a Luer rongeur or bone osteotome, partial resection of the spinous process was performed with detachment of the interspinous and supraspinous ligaments at the operated level with exposure if the lamina bilaterally. Under surgical microscope bilateral partial laminectomy was performed (even if symptoms were mainly unilateral) with partial facetectomy (up to 50% of the facet joint as required for adequate decompression at the surgeon’s discretion) using a 4.5-mm high-speed burr or a Kerrison rongeur. No discectomy or annulotomy was performed. The ligamentum flavum was then completely resected to expose the transversing nerve roots. A fine hook was used to assure there is no residual pressure on the nerve root in the region of its shoulder, axilla and the neuroforamen and that the nerve root is freely mobilized.

#### Minimally invasive approach

A tubular system (METRx Medtronic company, Minneapolis, Minnesota, USA) was utilized. The procedure was performed as described by Alimi et al. [15] with the exception of performing decompression of both sides through a unilateral port. A tube was inserted at the affected side (in case of bilateral symptoms at the more affected side), and bilateral decompression was achieved by a tilt of the operating table and angulation of the surgical microscope for contralateral decompression. In case of bilateral symptoms, laminotomy, partial facetectomy (up to 50% of the facet joint as required for adequate decompression at the surgeon’s discretion) and flavectomy were performed bilaterally as needed to release the pressure. In case of unilateral symptoms, bone resection was performed only at the symptomatic side, whereas at the asymptomatic side only flavectomy was performed (without bone resection). The spinous process and the posterior ligamentous complex were left intact.

Postoperatively, all patients were allowed to mobilize as pain allowed and most patients were discharged from the hospital at the first postoperative day. Isometric strengthening and exercise program were initiated three weeks postoperatively. Patients were followed up in our outpatient clinic at 6-week, 3-month, 6-month and 1-year time.

All patients who presented postoperatively with new-onset radicular symptoms, not improving with analgesia and anti-inflammatory medication within few weeks, were referred to lumbar MRI with contrast material in query of lumbar nerve root compression explaining the symptoms.

### Data analysis

Continuous parameters are presented as the means and standard deviations and categorical parameters as proportions. Age and BMI were compared between the groups using independent two-tailed *t* test. Timing of postoperative disc herniation was compared between the groups using Mann–Whitney test. Gender, affected level and disc herniation rate were compared using the Fisher’s exact test. All statistical analyses were performed using MedCalc Statistical Software version 19.7.2 (MedCalc Software Ltd, Ostend, Belgium). A *p*-value of < 0.05 was considered significant.

## Results

A total of 563 patients with a minimal follow-up of 1 year were available for analysis (follow-up rate of 85%), 326 were underwent mini-open decompression, and 237 underwent minimally invasive decompression utilizing a tubular system. Patients who underwent mini-open surgery were slightly older than patients who underwent minimally invasive surgery (65 ± 10 years vs. 62 ± 12 years, respectively, *p* = 0.002) and had slightly lower BMI (28 ± 4 kg/m^2^ vs. 30 ± 5 kg/m^2^, respectively, *p* = 0.01). Gender distribution, operated level and the number of operated segments were similar between the groups. Patients’ demographics and clinical data are summarized in Table 1.

**Table 1:** Demographics and clinical data of the study groups

	Total cohort (*N* = 563)	Mini-open partial laminectomy (*N* = 326)	Minimally invasive laminotomy (*N* = 237)	*p* value*
*Gender*				
Female	268 (48%)	147 (45%)	121 (51%)	0.16
Male	295 (52%)	179 (55%)	116 (49%)	
Age (years)	64 ± 12 (45 to 75)	65 ± 10 (45 to 72)	62 ± 12 (48 to 75)	0.002
BMI (kg/m^2^)	29 ± 5 (21 to 34)	28 ± 4 (21 to 32)	30 ± 5 (23 to 34)	0.01
*Operated segments*				
Single level	485 (86%)	287 (88%)	198 (83%)	0.12
L1–L2	4 (1%)	2 (1%)	2 (1%)	0.74
L2–L3	29 (5%)	20 (6%)	9 (4%)	0.21
L3–L4	61 (11%)	39 (12%)	22 (9%)	0.31
L4–L5	293 (52%)	174 (53%)	119 (50%)	0.46
L5–S1	98 (17%)	52 (16%)	46 (19%)	0.28
Multi-level	78 (14%)	39 (12%)	39 (17%)	0.14
Two levels	62 (11%)	32 (10%)	30 (13%)	0.29
Three levels	12 (2%)	4 (1%)	8 (3.5%)	0.08
Four levels	4 (1%)	3 (1%)	1 (0.5%)	0.48

Gender and operated segments values are presented as *n* (% of values in relation to each designated group). Age and BMI values are presented as mean ± standard deviation (range)

*Gender and operated segments ratios were compared between the mini-open and minimally invasive groups using Fisher’s exact test. Age and BMI values were compared using unpaired two-tailed *t* test

Forty-five patients were referred postoperatively to lumbar MRI due to acute radicular symptoms not improving with analgesia and anti-inflammatory medication within few weeks; 21 of them (3.7% from the total cohort) were diagnosed with acute postoperative disc herniation at the operated level. None of these patients had retrolisthesis. The rate of postoperative disc herniation was found to be significantly higher in the mini-open group with 19 of 326 cases (5.8%) compared to 2 of 237 cases (0.8%) in the minimally invasive group (*p* = 0.002). Postoperative disc herniation rate was also found to be significantly higher following multi-level operation (8 of 78 cases; 10.2%) compared to single-level operation (13 of 485 cases; 2.7%) (*p* = 0.004). A higher rate of disc herniation was found following mini-open surgery compared to minimally invasive surgery in both multi-level procedures (20.5% vs. 0%, respectively, *p* = 0.003) and single-level procedures (3.8% vs. 1.0%, respectively, *p* = 0.049). The presentation of postoperative new-onset radicular symptoms, representing the timing of disc herniation, occurred earlier following mini-open surgery compared to minimally invasive surgery (80 ± 6 days vs. 39 ± 16 days, respectively, *p* = 0.023). Most postoperative disc herniations following a single-level operation (10 out of 13, 76.9%), occurred at L4–L5 level. Comparison of postoperative disc herniating rate and timing is summarized in Table 2.

**Table 2:** Cases of postoperative disc herniation among the study groups

	Total cohort	Mini-open partial laminectomy	Minimally invasive laminotomy	*p* value*
Postoperative disc herniation (overall)	21 of 563 (3.7%)	19 of 326 (5.8%)	2 of 237 (0.8%)	0.002
*Timing of disc herniation (onset of radicular symptoms) (days)*				
	42 ± 20 (19 to 84)	39 ± 16 (19 to 71)	80 ± 6 (76 to 84)	0.023
*Postoperative disc herniation after a single-level operation*				
Overall	13 of 485 (2.7%)	11 of 287 (3.8%)	2 of 198 (1.0%)	0.049
L1–L2	0	0	0	
L2–L3	0	0	0	
L3–L4	1	1	0	
L4–L5	10	8	2	
L5–S1	2	2	0	
*Postoperative disc herniation after a multi-level operation*				
Overall	8 of 78 (10.2%)	8 of 39 (20.5%)	0 of 39 (0.0%)	0.003
Two levels	7	7	0	
Three levels	1	1	0	
Four levels	0	0	0	

Values are presented as *n* (% of values in relation to each designated group)

*Comparisons between the mini-open and minimally-invasive groups were performed using Fisher’s exact test. Comparison of timing of disc herniation was performed using Mann–Whitney test

**Fig. 1:**
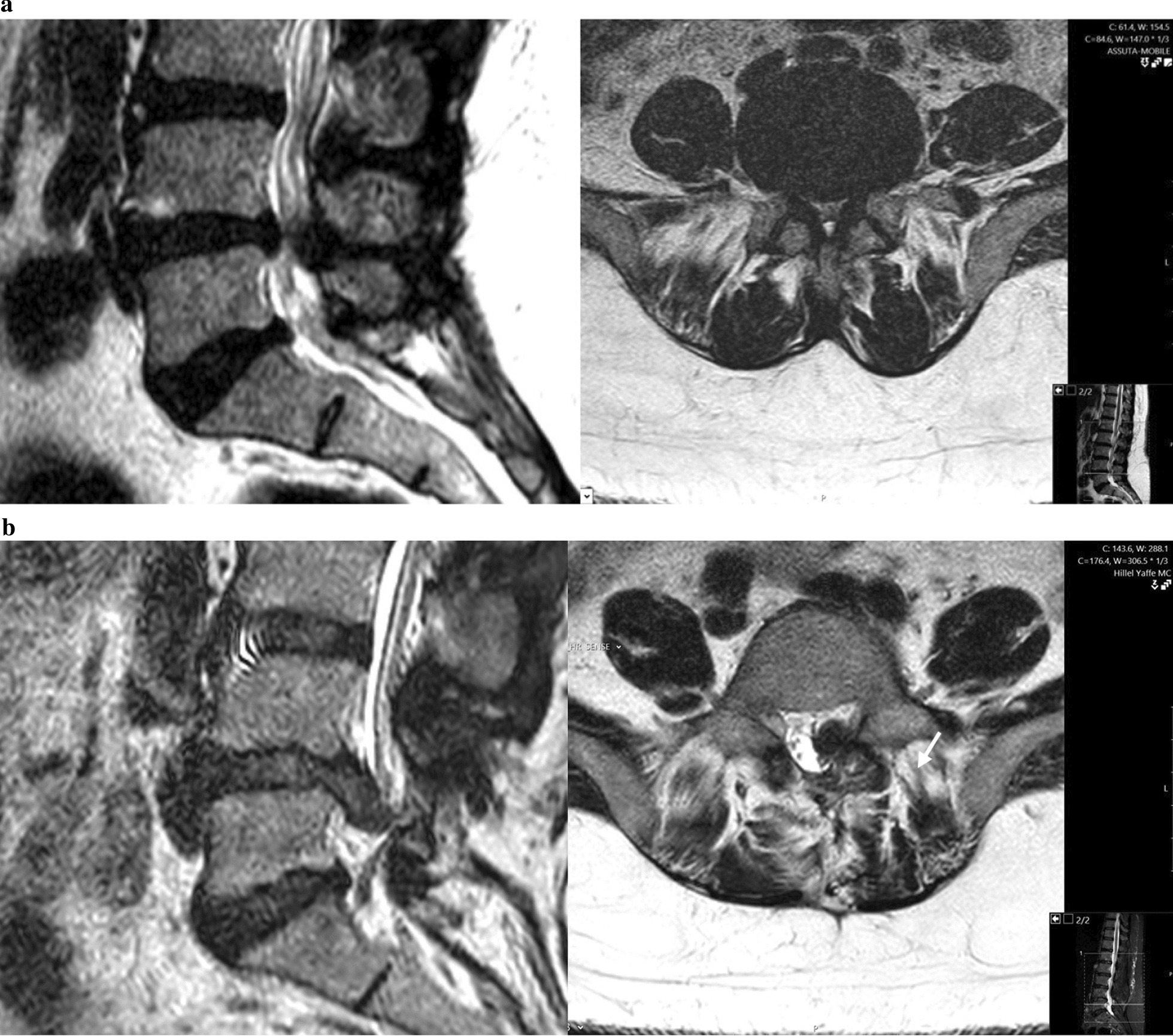
**a** Preoperative lumbar MRI images of a 62-year-old male with chronic bilateral L5 radicular pain and neurological claudication due to severe L4–5 spinal stenosis. **b** Postoperative MRI images of the same patient following mini-open decompression (involving bilateral partial laminectomy) demonstrating acute paracentral disc herniation

Six patients of those who were diagnosed with acute disc herniation following mini-open decompression underwent further surgery for disc removal (MRI images of one of these patients are presented in Fig. 1) compared to none of those who were diagnosed with acute disc herniation following minimally invasive decompression (6 of 19 patients vs. 0 of 2 patients, respectively; *p* = 0.98). The remaining patients were treated non-operatively.

## Discussion

Decompression surgery for patients suffering from moderate to severe lumbar spinal stenosis (LSS) refractory to non-operative treatment is a common practice [3, 4]. Many studies compared the outcome of open and minimally invasive techniques for decompression of LSS and failed to demonstrate clinical superiority of one technique over the other [5–7]. However, open lumbar decompression techniques, which commonly involve a wider posterior bone resection and ligament detachment, were associated with higher motion at the operated segment and increased disc annulus pressure representing relative segmental instability, compared to minimally invasive decompression techniques [8–11]. The intervertebral disc at the affected level of patients undergoing LSS is commonly degenerated with frail annular ring prone to bulging or tearing [1, 2]. The combination of degenerated intravertebral disc at the affected level and relative segmental instability following surgical decompression of LSS may pose a higher risk of postoperative disc herniation [12]. The purpose of our study was to compare the rate of acute symptomatic disc herniation following mini-open compared to minimally invasive decompression of LSS. To our knowledge, our study is the first to directly compare the incidence of disc herniation following mini-open versus minimally invasive LSS decompression.

The overall incidence of acute disc herniation following LSS decompression in our cohort was 3.7% (21 of 563 patients) with significantly lower incidence following minimally invasive procedures (2 of 237 cases; 0.8%) compared to mini-open procedures (19 of 326 cases; 5.8%) (*p* = 0.002). A relative segmental instability at the affected level in the mini-open group, related to greater amount of bone resection and soft tissue damage, may explain this difference.

Takenaka et al.[12] reviewed 381 patients who underwent open bilateral partial laminectomy for the treatment of LSS and reported on 18 cases (4.7%) of acute symptomatic disc herniation within 2-year follow-up; four of them required further surgery (the timing of disc herniation after the surgery was not reported). This rate of disc herniation following open decompression of LSS seems slightly lower compared to our finding of 5.8%, perhaps due to more extensive bone resection in our cohort, which included partial facetectomy (not described by Tekenaka’s et al.) known as a risk factor of segmental instability [10]. Minamide et al. [13] reviewed 310 patients who underwent minimally invasive laminotomy (including partial facetectomy as required) for LSS, of them 4 patients (1.3%) developed increased disc bulging/disc herniation at the affected level (all treated non-operatively), similar to our finding of 0.8%. Although facetectomy increases to risk of segmental instability, we believe that partial facetectomy, kept to the minimum required, has an important role in adequate decompression of LSS (whether mini-open or minimally invasive). Our results suggest that minimally invasive decompression of LSS (sparing the spinous process and posterior ligament complex) may minimize the risk for postoperative acute disc herniation even when partial facetectomy is performed.

Not surprisingly, the higher rate of postoperative disc herniation among patients who underwent mini-open decompression was noticeably higher following multi-level procedures (8 of 39 cases; 20.5%) compared to single-level procedures (11 of 287; 3.8%) (*p* = 0.001), suggesting that the more extensive nature of multi-level mini-open surgery, associated with relative segmental instability, poses a greater risk of acute postdecompression disc herniation. Also, the presentation of radicular symptoms, related to acute postoperative disc herniation, appeared earlier following mini-open procedures (averaged 39 days postoperatively) compared to minimally invasive procedures (averaged 80 days postoperatively) (*p* = 0.02), perhaps due to the relative higher segmental instability associated with these procedures.

Six of our 19 patients who were diagnosed with acute disc herniation following mini-open decompression underwent further surgery for disc removal, compared to 4 of 18 patients reported by Takenaka et al. [12]. Considering the relatively small number of patients these findings seem similar. The bilateral decompression that was routinely performed in all cases during the index surgery may played a role in minimizing patients’ symptoms related to the acute disc herniation.

### Limitations

Our study has several limitations. Firstly, the retrospective setup of the study has inherent limitations; however, we believe that relatively large cohort minimizes selection bias and other cofounders. Secondly, only patients with postoperative radicular symptoms not improving with medication were referred to MRI investigation; therefore, the actual incidence of postoperative disc herniation (including asymptomatic cases) remains unknown and may be higher than the overall 3.7% found in our cohort. Finally, we had a relatively short follow-up of only one year; however, since all postdecompression disc herniations in our cohort were diagnosed during the first 3 months after the index surgery, we believe that a 1-year follow-up is sufficient for the purpose of our study.

## Conclusion

The incidence of postoperative disc herniation following decompression of symptomatic lumbar spinal stenosis was 5.8% after mini-open bilateral partial laminectomy compared to only 0.8% after minimally invasive laminotomy (*p* = 0.002). These findings highlight the more extensive nature of open surgery associated with relative segmental instability that poses a greater risk for postoperative disc herniation. Further prospective high-quality clinical trials are required to evaluate the actual incidence of this complication in order to optimize patient care.

## Data Availability

The datasets used and/or analyzed during the current study are available from the corresponding author on reasonable request.
